# Are Nutrition-Related Knowledge and Attitudes Reflected in Lifestyle and Health Among Elderly People? A Study Across Five European Countries

**DOI:** 10.3389/fphys.2018.00994

**Published:** 2018-07-31

**Authors:** Marta Jeruszka-Bielak, Anna Kollajtis-Dolowy, Aurelia Santoro, Rita Ostan, Agnes A. M. Berendsen, Amy Jennings, Nathalie Meunier, Anna Marseglia, Elodie Caumon, Rachel Gillings, Lisette C. P. G. M. de Groot, Claudio Franceschi, Sophie Hieke, Barbara Pietruszka

**Affiliations:** ^1^Department of Human Nutrition, Warsaw University of Life Sciences-SGGW, Warsaw, Poland; ^2^Department of Experimental, Diagnostic and Specialty Medicine, University of Bologna, Bologna, Italy; ^3^C.I.G. Interdepartmental Centre “L. Galvani”, University of Bologna, Bologna, Italy; ^4^Division of Human Nutrition and Health, Wageningen University & Research, Wageningen, Netherlands; ^5^Norwich Medical School, University of East Anglia, Norwich, United Kingdom; ^6^Centre Hospitalier Universitaire de Clermont Ferrand, Clermont-Ferrand, France; ^7^Care Sciences and Society, Department of Neurobiology, Aging Research Center, Karolinska Institutet and Stockholm University, Stockholm, Sweden; ^8^Institute of Neurological Sciences (IRCCS), Bologna, Italy; ^9^European Food Information Council, Brussels, Belgium

**Keywords:** nutrition-related knowledge, nutrition-related attitudes, lifestyle, health, the elderly

## Abstract

**Background:** Nutrition-related knowledge (NRK) and nutrition-related attitudes (NRAs) are necessary for dietary changes toward healthier dietary patterns. In turn, healthier dietary patterns can be beneficial in maintaining health of older adults. Therefore, the aim of this cross-sectional study was to investigate whether NRK and NRAs were associated with lifestyle and health features among older adults (65+ years) from five European countries (France, Italy, Poland, the Netherlands and United Kingdom).

**Methods:** Within the European project NU-AGE, 1,144 healthy elderly volunteers (65–79 years) were randomly assigned to two groups: intervention (NU-AGE diet) or control. After 1-year of follow-up, both NRK and NRAs were assessed during exit interviews, in combination with a number of lifestyle and health variables (e.g., physical activity, smoking, alcohol use, BMI, self-assessed health status). Multivariable linear regression models were used in data analysis.

**Results:** In the NU-AGE study sample, good NRK was associated with lower BMI and higher physical activity. More positive NRAs were related to lower BMI and self-reported very good or good appetite. Moreover, both NRK and NRAs were associated with some socio-economic determinants, like financial situation, age, education, living area (for NRK), and country (for NRAs). Participants in the intervention group showed a better NRK (β = 0.367 [95% CI: 0.117; 0.617], *p* = 0.004) and more positive NRAs (β = 0.838 [95% CI: 0.318; 1.358], *p* = 0.002) than those in the control group. Higher self-evaluated knowledge was also significantly related to more positive NRAs (*p* < 0.001). The most popular sources of nutrition information were food labels, books and magazines on health, the dietitian and the doctor's office, although their importance varied significantly among countries, and, to a lesser extent, between women and men and between intervention and control group.

**Conclusion:** Higher NRK and NRA scores were associated with lower BMI and higher physical activity level. Therefore, a good nutrition-related knowledge and positive nutrition-related attitudes can strongly and positively influence the health status and quality of life among the older population. These results offer a great opportunity for policy makers to implement educational programs in order to counteract the epidemic of obesity and to improve the health span of European population.

## Introduction

The risk of various illnesses and disabilities increases with age what points out the importance of adequate nutritional intake for elderly people (Dean et al., 2009). Inadequate nutrition (deficiencies or excesses of nutrients) can lead to a series of body dysfunctions such as decreased immunity, frailty, and a number of noncommunicable diseases (NCDs). The most common NCDs in Europe are cardiovascular diseases (CVD; i.e., atherosclerosis, ischemic heart disease, and cerebrovascular disease) which altogether account for 35% of all death causes (The European Health Report, 2012).

Overweight and obesity are unhealthy conditions that significantly increase the risk of CVD, especially heart diseases and stroke, diabetes, certain cancers, and mortality (von Ruesten et al., 2011). Currently, about 39% of adults is overweight and 13% is obese, and these percentages are expected to increase in the coming years (WHO, 2017). However, overweight and obesity are modifiable conditions that can be prevented with lifestyle interventions aimed to improve dietary habits and physical exercise.

One of such interventions is the NU-AGE project which a main goal was to assess if a 1-year adherence to dietary recommendations based on Mediterranean-like dietary pattern can reduce inflammageing, optimize health and quality of life in European older adults (Berendsen et al., 2014). Inflammageing, meaning the chronic low-grade inflammatory status resulted from age (Franceschi et al., 2000), is considered to be involved in developing many age-related chronic diseases and geriatric syndromes (Franceschi et al., 2000; Cevenini et al., 2013).

The Mediterranean diet due to its antioxidant, anti-inflammatory and prebiotic properties can counteract inflammageing and reduce risk of developing NCDs, including CVD (Chatzianagnostou et al., 2015; Veronese et al., 2017). Typical Mediterranean foods contain nutritional hormetins that are able to activate specific stress-response pathways, among others: (1) phytochemicals (e.g., phenolic antioxidants, terpenoids, carotenoids, and allium derived sulfur compounds) present in olives, legumes, leafy green vegetables, tomatoes, garlic, and onion which activate nuclear factor erythroid 2 (Nrf2); (2) resveratrol present in grapes and red wine which regulates redox homeostasis, activates Nrf2 and sirtuin pathway, and blocks nuclear factor κB (NF-κB); (3) n-3 polyunsaturated fatty acids present in fish and nuts which activate Nrf2 and block NF-κB; (4) fiber present in legumes, unrefined whole-grain cereals, fresh vegetables, and fruits which cooperates with cellular stress pathways (Martucci et al., 2017).

Nutrition-related knowledge (NRK) and nutrition-related attitudes (NRAs) concern the individual's ability to understand food-related and nutrition-related terminology as well as the attitudes (emotions, motivations, perceptions, and cognitive beliefs) around the person's eating behavior toward food (Macías and Glasauer, 2014). Significant positive relationships between NRK and dietary intake and/or between NRAs and dietary intake were reported in a meta-analysis (Axelson et al., 1985) and in a more recent systematic review (Spronk et al., 2014), although the associations were quite weak. As many intrinsic and extrinsic factors influence nutritional behavior, NRK and NRAs may not be sufficient but they are necessary for dietary improvements (Macías and Glasauer, 2014).

NRK and NRAs can be influenced by a range of factors. High educational attainment has been linked to a better nutritional awareness in older people (Moore et al., 1992; Lin and Lee, 2005; Shatenstein et al., 2008; De Vriendt et al., 2009). Sex of respondents is another differentiating factor of NRK (Olson et al., 1982; Nichols et al., 1988; Hickman et al., 1993). A number of studies showed that old women have higher NRK than men (Hickman et al., 1993; Parmenter et al., 2000; Hendrie et al., 2008; Lin et al., 2011; Shatenstein et al., 2013), but other studies have shown the opposite (Trent, 1992; Medeiros et al., 1993; Lin and Lee, 2005). Furthermore, age has been discussed as a discriminating factor. Elderly people seem to have lower NRK than their younger counterparts (Parmenter et al., 2000; Lin and Lee, 2005; Hendrie et al., 2008). Lastly, lifestyle factors such as smoking and alcohol consumption are also related to NRK or NRAs (Mukamal et al., 2003). For example, people with a positive attitude toward nutrition are more likely to be non-smokers (Mukamal, 2006). A better-perceived appetite is also associated with positive attitudes toward nutrition (Shatenstein et al., 2013). Organoleptic properties (e.g., taste, smell, color, and food consistency) are the basic food selection factors (Moore et al., 1992; Backman et al., 2002; Cooke, 2004). Decreased perception of organoleptic features in older adults contributes to a decreased appetite (Shahar et al., 2003).

Older consumers are at risk of poor nutrition and diet-related diseases, therefore, it is very important to introduce effective educational and intervention programs to promote better health status of the elderly. It also means that we need a better understanding of what older consumers think, how they make decisions about food, nutrition and health, and which sources they trust most.

In this cross-sectional study, we aim to investigate how NRK and NRAs are associated with lifestyle and health conditions in the NU-AGE elderly participants from five countries (France, Italy, Poland, the Netherlands and United Kingdom). Additionally, we examine the sources of nutrition information to find those most trustworthy in the opinion of the NU-AGE study sample.

## Materials and methods

### Participants

The European NU-AGE study was carried out in five countries (France, Italy, Poland, the Netherlands and United Kingdom). Healthy European men and women aged 65–79 years, free of clinically diagnosed overt disease for at least 2 years, free living and independent were included in the NU-AGE baseline examination. Exclusion criteria were: overt disease such as aggressive cancer or dementia; unstable organ failure or organ failure necessitating a special diet; heart, renal, respiratory or liver failure; type 1 diabetes mellitus; chronic use of corticosteroids; recent (previous 2 months) use of antibiotics; recent (previous 3 months) change to habitual medication (e.g., statins and thyroxine) use; malnutrition, as diagnosed by body mass index <18.5 kg/m^2^; body weight loss of >10% within 6 months; presence of frailty (as assessed by the presence of at least three out of five criteria: unintentional weight loss, self-reported exhaustion [weakness (grip strength), slow walking speed, and low physical activity] (Fried et al., 2001).

At baseline (April 2012–January 2014), volunteers were randomly assigned into two groups, intervention (NU-AGE diet) or control. Participants randomized into the NU-AGE diet group received monthly counseling from a trained dietician/research nutritionist and individually tailored dietary advice based on Mediterranean dietary pattern (Berendsen et al., 2014), as well as a selection of products that could contribute to a healthier diet, e.g., whole-grain pasta, low fat and low sodium cheese, olive oil, margarine rich in mono- and polyunsaturated fatty acids, and a vitamin D supplement. Participants in the control group received current national dietary recommendations available in each country. After 1-year follow-up, all participants were asked to fill in a Nutrition Knowledge Questionnaire and the General Questionnaire including the questions on NRAs. A total of 1,144 older participants completed the follow-up examination and were used as a study population for the present study.

The NU-AGE study is registered at ClinicalTrials.gov, identifier NCT01754012. All subjects gave written informed consent in accordance with the Declaration of Helsinki. The study protocol was approved by ethics committees in each country. More details on the NU-AGE study are given elsewhere (Santoro et al., 2014).

### Data collection

Information on socio-demographics (i.e., age, sex, education, marital status, living area, and financial situation), lifestyle (i.e., smoking, alcohol consumption, physical activity, number of usual meals), and health conditions were collected by means of standardized questionnaires. Age was categorized into two groups: ≤ 75 and > 75 years of age. Education was categorized into elementary, secondary, and college/university based on years of education. Physical activity was assessed by the Physical Activity Scale for the Elderly (PASE), (Washburn et al., 1993) and grouped into quartiles. Participants' self-reported levels of physical activity and knowledge about healthy food and healthy eating as well as number of meals eaten daily were used.

Body Mass Index (BMI) was calculated on the basis of measured values of weight and height, as weight in kilogram divided by height in meter square. BMI results were categorized into underweight (<18.5 kg/m^2^), normal body weight (18.5–24.9 kg/m^2^), overweight (25.0–29.9 kg/m^2^), or obese (≥30.0 kg/m^2^), according to the WHO classification (WHO, 1998).

Mini-Nutritional Assessment (MNA) (Guigoz et al., 1996), Mini-Mental State Examination (MMSE) (Folstein et al., 1975), Activities of Daily Living (ADL) (Katz, 1983), and Instrumental Activities of Daily Living (IADL) (Katz, 1983) were used as measures of physical and cognitive health status. For MNA, cut-off ≤ 11 points for possible undernutrition was applied (Cuervo et al., 2008). MMSE score in the range of 24–30 points was used as a normal cognitive mental status (Folstein et al., 1975). Exhaustion was measured by two questions (“I felt that everything I did was an effort” and “I could not get going”) from the Center for Epidemiologic Studies Depression Scale (CES-D Scale) (Radloff, 1977) and was stated if at least one condition was present for at least 3 days in the previous week. Number of chronic diseases was calculated on the current existence of diseases reported by participants and was divided into five categories: 0 (no chronic diseases were reported), 1, 2, 3, and 4 or more diseases. Participants' self-evaluated health status and appetite were also used as health-related determinants.

### Assessment of nutrition-related knowledge and attitudes

NRK data were collected using the Nutrition Knowledge Questionnaire (adapted from the Food Labelling to Advance Better Education for Life project), which included 15 items on general nutrition knowledge (e.g., recommendations on calories, fluids, and selected nutrients intake, as well as sources of selected nutrients). All items were closed-ended, four were dichotomous and 11 were multiple choice with four, five or six options to choose from. A score of 1 was assigned to a good response and 0 otherwise. The score of NRK was computed as the sum of the correctly answered items ranging from 0 to 15 points. Higher score indicated better nutritional knowledge.

NRAs were assessed by five statements, related to everyday diet and food choices, on a seven-point Likert scale (range = 1–7; response options ranged from “strongly disagree” to “strongly agree”). The five statements were: (1) “The healthiness of food has little impact on my food choices”, (2) “It is important for me that my daily diet contains a lot of vitamins and minerals”, (3) “I always follow a healthy and balanced diet”, (4) “I do not avoid foods, even if they may raise cholesterol”, and (5) “I eat what I like and I do not worry much about the healthiness of food”. Three of five statements (No. 1, 4, 5) had inverse response scale, and thus were recoded accordingly. The scores from the five items were summed up and a total score (range = 5−35 points) was obtained. Higher score meant more positive attitude for healthy foods and diet. Six respondents were removed from further analysis for NRAs because they skipped at least one of these five statements.

Based on the distribution, the continuous scores of NRK and NRAs were divided into tertiles. NRK was categorized into “Insufficient” (I tertile), “Quite good” (II tertile) and “Good” (III tertile). NRAs were grouped into “Negative” (I tertile), “Neutral” (II tertile), and “Positive” (III tertile).

Data on sources of nutrition information were also obtained by the Nutrition Knowledge Questionnaire. Participants were asked to indicate in a closed-ended question with 13 options all sources of nutrition information that they used. In another question they could also indicate a maximum of three sources out of those selected earlier, that were most reliable for them.

### Statistical analysis

Chi-square test was used to examine differences in socio-demographic, lifestyle and health determinants by the tertiles of NRK and NRAs.

Multivariable stepwise linear regression models, with backward elimination, were conducted to assess the associations of NRK or NRAs (entered as continuous variables) with all socio-demographic, lifestyle, and health covariates. Age and BMI were entered into the models as continuous variables while all others as categorical ones. R-squared were used to compare the goodness-of-fit for each model. Results are presented as unstandardized beta with 95% confidence interval (CI) and standardized beta coefficients.

For all 13 sources of nutrition information, the total number of indications was calculated and compared among categories for gender, group, and country with the Chi-square test. Results are shown as percentage of indications, i.e., the number of responses of each source in relation to the number of people in a given category.

For all analysis, the significance level was set at 0.05. The statistical analyses were performed using the Statistical Package for the Social Sciences, version 23.0 (SPSS, Chicago, IL, USA).

## Results

### Characteristics of study population

The characteristics of study population are presented in Table 1. Of the 1,144 participants, 635 (56%) were women, well-educated (56% completed the secondary school and 46% graduated from college or university), living with spouse or partner (67%), mostly in cities (55%). Most of the participants reported no financial difficulties; their physical activity was of moderate or light intensity (40% and 38%, respectively). According to a declaration, about 69% of participants consumed three meals a day.

**Table 1:** Characteristics of NU-AGE study population and nutrition-related knowledge (NRK) and nutrition-related attitudes (NRAs) according to socio-demographic and lifestyle determinants among NU-AGE participants (*n* = 1,144).

**Characteristics**	**Total n (%)**	**NRK n (%)**			***p*-value**	**NRAs n (%)**			***p*-value**
		**Insufficient (≤ 6 points)**	**Quite good (7–8 points)**	**Good (≥9 points)**		**Negative (≤ 24 points)**	**Neutral (25–28 points)**	**Positive (≥29 points)**	
**Age (years)**					0.001				0.829
≤ 75	974 (85.1)	296 (30.4)	347 (35.6)	331 (34.0)		355 (36.7)	290 (30.0)	323 (33.4)	
>75	170 (14.9)	75 (44.4)	51 (30.2)	43 (25.4)		64 (37.6)	47 (27.6)	59 (34.7)	
**Gender**					0.119				0.044
Female	635 (55.6)	199 (31.4)	211 (33.3)	224 (35.3)		215 (34.0)	188 (29.7)	230 (36.3)	
Male	507 (44.4)	171 (33.7)	186 (36.7)	150 (29.6)		203 (40.3)	149 (29.6)	152 (30.2)	
**Education**					0.014				0.144
Elementary	40 (3.5)	20 (50.0)	12 (30.0)	8 (20.0)^a^		10 (25.0)	13 (32.5)	17 (42.5)	
Secondary	571 (50.3)	186 (32.6)	213 (37.4)	171 (30.0)^a^		209 (36.7)	183 (32.1)	178 (31.2)	
College/University	524 (46.2)	162 (30.9)	169 (32.3)	193 (36.8)^b^		199 (38.0)	140 (26.7)	185 (35.3)	
**Group**					0.061				0.019
Intervention	573 (50.1)	174 (30.4)	193 (33.7)	206 (36.0)		187 (32.8)	178 (31.2)	205 (36.0)	
Control	570 (49.9)	197 (34.6)	204 (35.9)	168 (29.5)		232 (40.8)	159 (28.0)	177 (31.2)	
**Country**					< 0.001				0.021
Italy	243 (21.2)	96 (39.5)	98 (40.3)	49 (20.2)^a^^c^		72 (29.9)	67 (27.8)	102 (42.3)^a^	
UK	252 (22.0)	47 (18.7)	73 (29.0)	132 (52.4)^b^		88 (34.9)	76 (30.2)	88 (34.9)^a^^b^	
Netherlands	245 (21.4)	98 (40.2)	85 (34.8)	61 (25.0)^a^		86 (35.5)	79 (32.6)	77 (31.8)^a^^b^	
Poland	218 (19.1)	107 (49.1)	75 (34.4)	36 (16.5)^c^		91 (41.9)	64 (29.5)	62 (28.6)^b^	
France	186 (16.3)	23 (12.4)	67 (36.0)	96 (51.6)^b^		82 (44.1)	51 (27.4)	53 (28.5)^b^	
**Living area**					0.002				0.556
Urban	622 (54.7)	223 (35.9)	215 (34.6)	183 (29.5)^a^		229 (36.8)	178 (28.6)	215 (34.6)	
Rural	269 (23.6)	90 (33.5)	85 (31.6)	94 (34.9)^a^		107 (39.9)	79 (29.5)	82 (30.6)	
Suburban	247 (21.7)	56 (22.7)	94 (38.1)	97 (39.3)^b^		83 (33.6)	79 (32.0)	85 (34.4)	
**Marital status**					0.970				0.260
Never married or lived with partner	35 (3.1)	13 (37.1)	12 (34.3)	10 (28.6)		16 (45.7)	12 934.3)	7 (20.0)	
Married or living with partner	767 (67.3)	244 (31.8)	270 (35.2)	253 (33.0)		275 (35.9)	235 (30.7)	256 (33.4)	
Divorced, separated	143 (12.6)	45 (31.5)	48 (33.6)	50 (35.0)		49 (34.3)	45 (31.5)	49 (34.3)	
Widow/widower	194 (17.0)	67 (34.7)	65 (33.7)	61 (31.6)		79 (40.7)	45 (23.2)	70 (36.1)	
Financial situation^**^ **I/we:**					< 0.001				0.012
Have financial difficulties	66 (5.8)	24 (36.4)	29 (43.9)	13 (19.7)^a^^b^		32 (48.5)	13 (19.7)	21 (31.8)^a^	
Get by alright	379 (33.3)	149 (39.3)	138 (36.4)	92 (24.3)^a^		148 (39.1)	121 (31.9)	110 (29.0)^a^	
Manage quite well	355 (31.2)	105 (29.7)	133 (37.6)	116 (32.8)^b^		133 (37.5)	101 (28.5)	121 (34.1)^a^^b^	
Manage very well	310 (27.3)	83 (26.8)	84 (27.1)	143 (46.1)^c^		93 (30.0)	98 (31.6)	119 (38.4)^b^	
Do not know^*^	27 (2.4)	7 (25.9)	10 (37.0)	10 (37.0)		13 (48.1)	4 (14.8)	10 (37.0)	
**Current smoking**					0.693				0.085
Yes	39 (3.4)	15 (38.5)	13 (33.3)	11 (28.2)		16 (41.0)	16 (41.0)	7 (17.9)	
No	1099 (96.6)	354 (32.2)	382 (34.8)	362 (33.0)		403 (36.7)	320 (29.1)	375 (34.2)	
**Alcohol use**					< 0.001				0.016
Yes	906 (79.7)	273 (30.2)	305 (33.7)	327 (36.1)		328 (36.2)	288 (31.8)	290 (32.0)	
No	230 (20.3)	95 (41.3)	88 (38.3)	47 (20.4)		91 (39.6)	48 (20.9)	91 (39.6)	
**PASE**					0.005				0.077
I quartile (< 92.43)	284 (25.0)	115 (40.5)	92 (32.4)	77 (27.1)^a^		113 (39.8)	80 (28.2)	91 (32.0)	
II quartile (92.43–123.60)	285 (25.0)	91 (31.9)	87 (30.5)	107 (37.5)^b^		116 (40.7)	85 (29.8)	84 (29.5)	
III quartile (123.61–162.72)	284 (25.0)	83 (29.3)	100 (35.3)	100 (35.3)^bc^		83 (29.2)	89 (31.3)	112 (39.4)	
IV quartile (>162.73)	284 (25.0)	79 (27.8)	115 (40.5)	90 (31.7)^c^		106 (37.5)	82 (29.0)	95 (33.6)	
**Level of physical activity (self-evaluation)**					0.002				0.046
Sitting for most of the time	62 (5.4)	29 (47.5)	17 (27.9)	15 (24.6)^a^		25 (40.3)	22 (35.5)	15 (24.2)^a^^b^	
Light intensity exercise	434 (38.1)	156 (35.9)	149 (34.3)	129 (29.7)^a^^b^		173 (40.0)	133 (30.7)	127 (29.3)^a^	
Moderate intensity exercise (1–2 h/week)	456 (40.1)	137 (30.0)	171 (37.5)	148 (32.5)^bc^		158 (34.6)	121 (26.5)	177 (38.8)^b^	
Moderate intensity exercise (>3 h/week)	150 (13.2)	40 (26.7)	48 (32.0)	62 (41.3)^cd^		48 (32.0)	53 (35.3)	49 (32.7)^a^^b^	
Intense exercise regularly	37 (3.2)	7 (18.9)	10 (27.0)	20 (54.1)^d^		15 (40.5)	8 (21.6)	14 (37.8)^a^^b^	
**Number of meals (according to a declaration)**					0.002				0.164
< 1 meal a day^*^	2 (0.2)	0	0	2 (100.0)		1 (50.0)	1 (50.0)	0	
One meal a day	24 (2.1)	7 (29.2)	13 (54.2)	4 (16.7)^a^^b^		13 (54.2)	5 (20.8)	6 (25.0)	
Two meals a day	131 (11.5)	36 (27.5)	48 (36.6)	47 (35.9)^a^		60 (45.8)	35 (26.7)	36 (27.5)	
Three meals a day	789 (69.3)	249 (31.6)	259 (32.9)	280 (35.5)^a^		278 (35.2)	240 (30.4)	271 (34.3)	
More than three meals a day	192 (16.9)	76 (39.6)	75 (39.1)	41 (21.4)^b^		67 (34.9)	56 (29.2)	69 (35.9)	
**Knowledge about healthy food and healthy eating (self-evaluation)** ^***^					0.001				< 0.001
Not knowledgeable	17 (1.5)	10 (58.8)	5 (29.4)	2 (11.8)^a^		8 (47.1)	8 (47.1)	1 (5.8)^a^^b^	
Rather not knowledgeable	53 (4.7)	23 (43.4)	12 (22.6)	18 (34.0)^a^		31 (58.5)	11 (20.8)	11 (20.8)^a^	
Neither knowledgeable nor not knowledgeable	174 (15.3)	67 (38.3)	65 (37.4)	42 (24.1)^a^		91 (52.3)	45 (25.9)	38 (21.8)^a^	
Rather knowledgeable	590 (51.8)	193 (32.8)	202 (34.3)	194 (32.9)^a^		208 (35.3)	195 (33.1)	187 (31.7)^b^	
Knowledgeable	304 (26.7)	75 (24.7)	111 (36.5)	118 (38.8)^b^		81 (26.6)	78 (25.7)	145 (47.7)^c^	

NRK was divided into tertiles: insufficient (≤ 6 points), quite good (7–8 points), good (≥9 points). NRAs was divided into tertiles: negative (≤ 24 points), neutral (25–28 points), positive (≥29 points).

a,b For each determinant values with different superscript are significantly different (p ≤ 0.05).

* Excluded from statistical analysis.

** Two categories “Have some financial difficulties” and “Have severe financial difficulties” were combined due to a low number of subjects (n = 4) in the second category.

*** *Categories “not knowledgeable at all” and “not knowledgeable” were combined into one category: “not knowledgeable,” similarly to last two categories “knowledgeable,” and “very knowledgeable” which created the category “knowledgeable,” due to low number of subjects in most extreme categories*.

After 1 year of follow-up, 45.5% of participants were overweight and 17% were obese. Five participants had BMI below 18.5 kg/m^2^ (Table 2). According to the MNA score, the majority of the population was not at risk of undernutrition and did not report exhaustion (89 and 88%, respectively). The highest proportion of participants perceived their appetite and their health status as good (55 and 52%, respectively), although 46% of them had at least four chronic diseases.

**Table 2:** Characteristics of NU-AGE study population and nutrition-related knowledge (NRK) and nutrition-related attitudes (NRAs) according to health determinants in NU-AGE participants (*n* = 1,144).

**Characteristics**	**Total n (%)**	**NRK n (%)**			***p*-value**	**NRAs n (%)**			***p*-value**
		**Insufficient (≤ 6 points)**	**Quite good (7–8 points)**	**Good (≥9 points)**		**Negative (≤ 24 points)**	**Neutral (25–28 points)**	**Positive (≥29 points)**	
**BMI (kg**·**m**^−2^**)** Underweight (< 18.5) Normal weight (18.5–24.9) Overweight (25–29.9) Obese (>30.0)	5 (0.4) 425 (37.2) 520 (45.5) 194 (16.9)	0 117 (27.5) 183 (35.2) 71 (36.8)	3 (60.0) 137 (32.2) 180 (34.6) 78 (40.4)	2 (40.0) ^a^^b^ 171 (40.2)^a^ 157 (30.2)^b^ 44 (22.8)^b^	< 0.001	2 (40.0) 124 (29.2) 202 (38.9) 91 (48.1)	2 (40.0) 123 (28.9) 157 (30.3) 55 (29.1)	1 (20.0)^a^^b^^c^ 178 (41.9)^a^ 160 (30.8)^b^ 43 (22.8)^c^	< 0.001
**MNA** Possible undernutrition (≤ 11 points) Not at risk for undernutrition (≥12 points)	124 (10.9) 1014 (89.1)	46 (37.1) 322 (31.8)	44 (35.5) 351 (34.6)	34 (27.4) 340 (33.6)	0.324	44 (35.5) 375 (37.0)	39 (31.5) 298 (29.4)	41 (33.1) 340 (33.6)	0.890
IADL score (max. 8 points)^*^ ≤ 5 points > 5 points	506 (44.4) 633 (55.6)	171 (33.8) 198 (31.3)	185 (36.6) 210 (33.2)	150 (29.6) 224 (35.4)	0.116	204 (40.4) 215 (34.0)	149 (29.5) 188 (29.7)	152 (30.1) 230 (36.3)	0.041
ADL score (max. 6 points)^**^ ≤ 5 points 6 points	105 (9.6) 1029 (90.4)	36 (34.3) 330 (32.1)	44 (41.9) 351 (34.1)	25 (23.8) 348 (33.8)	0.095	33 (31.4) 384 (37.3)	30 (28.6) 305 (29.6)	42 (40.0) 340 (33.0)	0.316
**Exhaustion (self-reported)** Yes No	131 (11.6) 1001 (88.4)	47 (35.9) 320 (32.0)	52 (39.7) 341 (34.1)	32 (24.4) 340 (34.0)	0.090	59 (45.0) 359 (35.9)	37 (28.2) 297 (29.7)	35 (26.7) 345 (34.5)	0.091
**Number of chronic diseases:** 0 1 2 3 4 or more	82 (7.2) 126 (11.0) 203 (17.7) 208 (18.2) 525 (45.9)	27 (32.9) 41 (32.5) 63 (31.0) 66 (31.9) 174 (33.1)	28 (34.1) 37 (29.4) 77 (37.9) 63 (30.4) 193 (36.8)	27 (32.9) 48 (38.1) 63 (31.0) 78 (37.7) 158 (30.1)	0.479	32 (41.6) 35 (27.8) 80 (39.4) 83 (39.9) 189 (36.1)	26 (33.8) 47 (37.3) 51 (25.1) 53 (25.5) 160 (30.5)	19 (24.7) 44 (34.9) 72 (35.5) 72 (34.6) 175 (33.4)	0.146
**Health status (self-evaluation)** Excellent Very good Good Fair/poor	88 (7.7) 266 (23.4) 588 (51.7) 196 (17.2)	21 (23.9) 85 (32.0) 184 (31.3) 78 (39.8)	28 (31.8) 67 (25.2) 226 (38.5) 74 (37.8)	39 (44.3)^a^ 114 (42.9)^a^ 177 (30.2)^b^ 44 (22.4)^c^	< 0.001	25 (28.4) 84 (31.6) 228 (38.8) 82 (41.8)	25 (28.4) 83 (31.2) 171 (29.1) 58 (29.6)	38 (43.2) 99 (37.2) 189 (32.1) 56 (28.6)	0.081
**Appetite (self-evaluation)** Poor/very poor Average Good Very good	16 (1.4) 240 (21.1) 628 (55.2) 254 (22.3)	5 (31.3) 88 (36.7) 197 (31.4) 78 (30.7)	9 (56.3) 83 (34.6) 208 (33.2) 95 (37.4)	2 (12.5) 69 (28.8) 222 (35.4) 81 (31.9)	0.151	8 (50.0) 112 (46.7) 230 (36.6) 69 (27.2)	5 (31.3) 65 (27.1) 184 (29.3) 83 (32.7)	3 (18.8)^a^^b^^c^ 63 (26.3)^a^ 214 (34.1)^b^ 102 (40.2)^c^	0.001

NRK was divided into tertiles: insufficient (≤ 6 points), quite good (7–8 points), good (≥ 9 points). NRAs was divided into tertiles: negative (≤ 24 points), neutral (25–28 points), positive (≥ 29 points).

a,b *For each determinant values with different superscript are significantly different (p ≤ 0.05)*.

* *Categories for IADL were based on variable distribution, where two picks were observed – first for the 5 points and the second – for 8 points*.

** *Categories for ADL were based on variable distribution; the majority (90.4%) got maximal score equaled 6 points, 9.1% got 5 points, and only one person got 2 points*.

Almost all participants (99%) had the MMSE score in the range of 24–30 points which indicated the lack of cognitive impairment (data not shown).

### Nutrition-related knowledge, lifestyle, and health-related factors

In the univariate analysis, NRK was significantly associated with the following factors: age, education, country of living, living area, financial situation, alcohol use, physical activity level, declared number of meals, BMI, and self-evaluated health status (Tables 1, 2). A significantly higher proportion of participants was classified to the third tertile (good NRK) when they were: of younger age (≤ 75 years); had higher level of education (college or university); were from the UK or Italy; lived in a suburban area; self-evaluated their financial situation as very good; had higher physical activity level (self-evaluated and PASE score); those who declared drinking alcohol as well as those with lower BMI, and excellent or very good perceived health status. Although there was no significant difference between intervention and control group for NRK tertiles (*p* = 0.061), intervention group scored a significantly higher mean of correct responses in nutrition test than the control group (51.7 ± 15.3% vs. 49.1 ± 15.0%; *p* = 0.003; data not shown).

The results of nutrition test were in line with the participants' self-evaluation of their knowledge about healthy food and healthy eating.

Results from multiple regression analysis show that most of determinants that were significant in relation to NRK in the univariate analysis remained significant also in the regression model (Table 3). Socio-demographic, lifestyle and health determinants, such as financial situation, age, education, living area, self-evaluated level of physical activity and BMI showed a significant independent association with NRK. Participants in the intervention group showed a better NRK (β = 0.367 [95% CI: 0.117; 0.617], *p* = 0.004) than those in the control group. Younger NU-AGE participants, with lower BMI, better financial situation, higher level of physical activity and education, living in suburban areas, and belonging to the intervention group showed higher NRK. These seven variables accounted for 12% of the variance in the NRK scores.

**Table 3:** Multiple regression analysis between socio-demographic, lifestyle and health and nutrition-related knowledge (NRK).

	**Nutrition-related knowledge**		
	**Unstandardized β (95% CI)**	**Standardized β**	***p-*value**
**Financial situation** Have financial difficulties Get by alright Manage quite well Manage very well	**0.395 (0.262; 0.527)** Ref. 0.208 (−0.352; 0.771) 0.672 (0.105; 1.240) 1.170 (0.592; 1.748)	**0.168** Ref. 0.044 0.137 0.230	<**0.001** Ref. 0.464 0.020 < 0.001
**Age**	−**0.086 (**−**0.118;** −**0.053)**	−**0.150**	<**0.001**
**BMI**	−**0.060 (**−**0.094;** −**0.027)**	−**0.104**	<**0.001**
**Education** Primary Secondary College/University	**0.400 (0.174; 0.626)** Ref. 1.093 (0.405; 1.780) 1.312 (0.617; 2.006)	**0.099** Ref. 0.241 0.289	**0.001** Ref. 0.002 < 0.001
**Level of physical activity** Sitting for most of the time Light intensity exercise Moderate intensity exercise (1–2 h/week) Moderate intensity exercise (>3 h/week) Intense exercise regularly	**0.221 (0.072; 0.369)** Ref. 0.455 (−0.129; 1.039) 0.562 (−0.027; 1.151) 0.808 (0.147; 1.466) 1.314 (0.423; 2.205)	**0.086** Ref. 0.098 0.122 0.120 0.103	**0.004** Ref. 0.126 0.061 0.016 0.004
**Group**			
Intervention Control	**0.367 (0.117; 0.617)** Ref.	**0.081** Ref.	**0.004** Ref.
**Living area** Urban Rural Suburban	**0.223 (0.066; 0.380)** Ref. −0.109 (−0.429; 0.211) 0.500 (0.178; 0.822)	**0.080** Ref. −0.020 0.091	**0.005** Ref. 0.503 0.002

*Multiple R = 0.350; Adjusted R^2^ = 0.123; F_(7, 1124)_ = 21.842; p < 0.001*.

*The values given in bold signify the main variables included into the model (7 variables)*.

### Nutrition-related attitudes, lifestyle, and health-related factors

In the univariate analysis, NRAs was significantly associated with the following socio-demographic and lifestyle determinants: gender, group, country, financial situation, alcohol use, and self-evaluated physical activity level (Table 1). Significant more positive NRAs were observed in: women; participants belonging to the intervention group; Italians (when compared to French and Polish people); subjects with very good financial situation; and those who declared no alcohol consumption. Besides, a positive association between NRAs and perceived knowledge about healthy food and eating was found – the proportion of participants in the third tertile increased with the increasing self-evaluated level of nutritional knowledge. Among health-related factors, NRAs were significantly associated with BMI, IADL score and self-evaluated appetite (Table 2). In particular, more positive NRAs were associated with a lower BMI; higher IADL score; very good or good appetite.

According to a multiple regression analysis, self-evaluated knowledge about healthy food and healthy eating showed the highest independent association with NRAs (Table 4). Other significant variables that affected NRA score were: BMI, self-evaluated appetite, country, and group. More positive NRAs were associated with higher self-evaluated nutritional knowledge, lower BMI, very good/good appetite, being Italian, and belonging to intervention group. Participants in the intervention group showed more positive NRAs (β = 0.838 [95% CI: 0.318; 1.358], *p* = 0.002) than those in the control group. Although in univariate analysis more variables were significantly related to NRAs, they did not remain significant in tested models, thus they were excluded from the final model. The best-fitted model accounted for 13% of the variance in NRA scores.

**Table 4:** Multiple regression analysis between socio-demographic, lifestyle and health and nutrition-related attitudes (NRAs).

	**Nutrition-related attitudes**		
	**Unstandardized β (95% CI)**	**Standardized β**	***p*-value**
**Nutrition knowledge about healthy food and healthy eating (self-evaluation)** Not knowledgeable Rather not knowledgeable Neither knowledgeable nor not knowledgeable Rather knowledgeable Knowledgeable	**1.285 (0.982; 1.589)** Ref. 0.352 (−2.093; 2.797) 0.685 (−1.544; 2.915) 2.626 (0.470; 4.783) 3.969 (1.782; 6.156)	**0.232** Ref. 0.016 0.052 0.275 0.368	<**0.001** Ref. 0.778 0.546 0.017 < 0.001
**BMI**	−**0.229 (**−**0.296;** −**0.163)**	−**0.188**	<**0.001**
**Appetite** **(self-evaluation)** Poor/very poor Average Good Very good	**0.884 (0.509; 1.258)** Ref. 1.334 (−0.927; 3.594) 2.238 (0.018; 4.458) 3.130 (0.862; 5.398)	**0.130** Ref. 0.114 0.233 0.273	<**0.001** Ref. 0.247 0.048 0.007
**Country** Italy UK Netherlands Poland France	−**0.419 (**−**0.608;** −**0.230)** Ref. −1.405 (−2.202; −0.609) −1.027 (−1.835; −0.218) −1.682 (−2.511; −0.854) −1.934 (−2.799; −1.068)	−**0.121** Ref. −0.122 −0.088 −0.139 −0.150	<**0.001** Ref. 0.001 0.013 < 0.001 < 0.001
**Group**			
Intervention Control	**0.838 (0.318; 1.358)** Ref.	**0.088** Ref.	**0.002** Ref.

*Multiple R = 0.368; Adjusted R^2^ = 0.135; F_(5, 1127)_ = 35.246; p < 0.001*.

*The values given in bold signify the main variables included into the model (5 variables)*.

### Sources of nutrition information

The most frequently indicated sources of nutrition information were food labels (60%), books and magazines on health (57%), dietitians (and other health professionals; 43%), the doctor's practice (42%), and Internet (39%) (Table 5). Significantly higher proportion of women than men pointed out books and magazines (61 vs. 49%), while the opposite situation was found for Internet (35 vs. 43%), and for family and friends (33 vs. 39%). After 1 year of follow-up, significant differences were noted between groups for food labels and dietitians, both being more often indicated by participants in the intervention group than in the control group (62 vs. 55% and 46 vs. 38%, respectively). Numerous significant differences were also observed among countries.

**Table 5:** Sources of nutrition information used/trusted by participants of the NU-AGE dietary intervention study (*n* = 1,144).

	**Total %**	**Gender**		**Group**		**Country**					**Most trusted sources**	
		**F %**	**M %**	**Inter-vention %**	**Control%**	**IT %**	**UK %**	**NL %**	**PL %**	**FR %**	**Total%**	**Order**
1. Food labels	59.8	61.0	56.1	62.5^a^	55.1^b^	53.9^ac^	71.1^b^	58.8^b^^c^	62.4^c^	44.6^b^^c^	26.0	V
2. Books, magazines on health	56.6	61.2^a^	48.9^b^	55.8	55.6	56.0^a^	40.2^b^	50.8^a^	75.2^c^	59.7^a^	48.9	II
3. Dietitian (and other health professionals)	42.9	44.7	39.1	45.8^a^	38.5^b^	43.2^a^^b^	43.8^a^^b^	35.4^a^	39.9^a^	50.5^b^	49.6	I
4. At doctor's practice	42.5	41.8	41.7	39.5	44.0	60.6^a^	34.9^b^	15.0^c^	54.6^a^	46.2^d^	41.7	III
5. Internet	39.3	35.5^a^	42.9^b^	36.8	40.7	29.5^ad^	45.0^b^^c^	44.6^b^^c^	39.9^cd^	33.3^d^	20.5	VI
6. Family and friends	36.6	33.3^a^	39.5^b^	34.2	37.8	30.7^a^	41.4^b^	29.2^a^	50.5^c^	28.0^a^	26.5	IV
7. Newspaper	34.0	33.1	33.5	31.9	34.8	35.3^a^	39.0^a^^b^	44.6^b^	19.3^c^	25.8^c^	12.4	VIII
8. Television	29.7	30.0	28.5	27.2	31.4	34.0	24.9	31.3	26.6	29.6	11.6	IX
9. At the pharmacy	25.4	26.3	23.2	25.3	24.5	29.9^a^	22.5^a^	15.4^b^	28.4^a^	30.1^a^	18.9	VII
10. General magazines	20.7	19.3	21.6	19.5	21.3	20.7	21.3	24.6	20.2	13.4	6.4	X
11. Radio	15.0	15.7	13.4	13.7	15.6	12.9^a^	24.5^b^	8.3^a^	13.8^a^	13.4^a^	4.8	XI
12. Supermarket magazines	14.6	15.5	13.0	14.2	14.6	14.1^a^	12.9^a^	27.9^b^	6.0^c^	9.1^ac^	3.2	XIII
13. Food manufactures (e.g., websites)	8.0	8.1	7.6	8.1	7.6	1.7^a^	6.4^b^	10.8^b^^c^	12.4^c^	8.6^b^^c^	3.5	XII

a,b *For each category values with different superscript are significantly different (p ≤ 0.05)*.

Finally, the most trusted sources of nutrition information were dietitians (50%), books and magazines on health (49%), and the doctor's practice (42%).

## Discussion

This survey examines whether NRK and NRAs are associated with lifestyle, health, and socio-demographic determinants among NU-AGE elderly participants from five European countries (France, Italy, Poland, the Netherlands and United Kingdom).

Higher NRK was associated with lower BMI, younger age, better financial situation, higher level of physical activity and education, living area (suburban areas respect to urban and rural areas), and group (intervention respect to control). More positive NRAs were associated with lower BMI, higher self-evaluated nutritional knowledge, very good/good appetite, being Italian, and belonging to intervention group.

### Nutrition-related knowledge

This study shows that BMI and self-evaluated level of physical activity, financial situation, age, education level, and living area had an independent and significant association with NRK. Interestingly, higher NRK was related to lower BMI and higher physical activity level. Similar results, namely negative association between NRK and BMI, and positive between NRK and physical activity level were found in adult Belgian women, although different methods for evaluation of physical activity were used (self-evaluation in the present study vs. short form of the International Physical Activity Questionnaire) (De Vriendt et al., 2009). On the contrary, according to Girois et al. (2001) BMI was not associated with the knowledge about fat and fiber either in Americans or Genevans aged 35–75 years.

There is a gap in literature to make the direct explanation of observed relationship between higher NRK (and more positive NRAs) and lower BMI but can be explained indirectly. Nutrition knowledge was significantly associated with higher adherence to a Mediterranean dietary pattern (Bonaccio et al., 2013), and in turn, healthy dietary patterns, including Mediterranean diet, were inversely related to BMI and/or prevalence of obesity (Schroder et al., 2004; Panagiotakos et al., 2006; Buckland et al., 2008; Veronese et al., 2017). The protective effect of diet, especially Mediterranean diet, against overweight/obesity can be attributed to (1) a large quantity of dietary fiber which increases satiety and satiation through mechanisms, such as prolonged mastication, increased gastric detention and enhanced release of cholecystokinin; (2) a low energy density and a low glycaemic load; (3) a high intake of monounsaturated fatty acids that have been found to improve glucose metabolism, and increase postprandial fat oxidation, as well as diet-induced thermogenesis (Buckland et al., 2008). However, other investigators did not observe the relationship between dietary quality or Mediterranean diet and body weight, or observed opposite tendencies, namely higher dietary quality like higher dietary diversity was associated with higher BMI (Bernstein et al., 2002; Azadbakht et al., 2006).

In the present study, older adults with better financial situation and higher education level showed better NRK. These findings are in line with other research, conducted in younger (Parmenter et al., 2000; Hendrie et al., 2008; De Vriendt et al., 2009) and elderly population (Lin and Lee, 2005). Australian adults who lived in area with “middle socio-economic status (SES)” scored significantly higher for overall knowledge and for three out of four sections of knowledge in comparison to subjects from “low SES” area (SES was derived from three attributes: income, educational attainment and unemployment in the areas). Education level was also an independent determinant of nutritional knowledge (Hendrie et al., 2008). In adult English population, both level of education and SES had significant and independent effects on NRK (Parmenter et al., 2000). It is quite unlikely that highly educated elderly became aware of concepts related to healthy nutrition during their scholastic/educational path since the majority of nutrition information are new and not taught at schools when these individuals were students. It can be argued that older adults who graduated from colleges or universities were more interested in health and nutrition information than they counterparts with lower education, and/or were also more capable in searching and remembering new knowledge. This hypothesis can be confirmed by significant differences among three categories of education level in sources of nutrition information. Internet was more frequently indicated by participants with highest education (44%) than those with secondary (35%) or primary education (15%). Opposite tendency was detected for supermarket magazines (10, 18, and 20%, respectively). Availability of Internet and computers, tablets, or smartphones may be also influenced by financial situation, what showed a strong impact on nutritional knowledge.

Age was another socio-demographic determinant that significantly influenced NRK. In particular, NRK decreased with increasing age and the finding is consistent with results obtained by Lin and Lee (2005) for elderly Taiwanese people. Other studies gave somewhat ambiguous results. For example, already mentioned Girois et al. (2001) did not observe such relation, although the population varied substantially in terms of age (from 35 to 75 years), while Parmenter et al. (2000) found the lowest NRK among the youngest (18–34 years) and the oldest (65 and over) subpopulations.

Subjects belonging to the NU-AGE intervention group showed a better NRK compared to the control group confirming the effectiveness of the NU-AGE dietary intervention. However, this conclusion should be made with some caution as the NRK was not evaluated at the baseline.

It is noteworthy that older adults who evaluated their health status as excellent or very good had significantly better NRK, while those with fair or poor health—the lowest. The association between health status and NRK (and NRAs) may be bidirectional, namely better health may be a final consequence of good NRK, NRAs (and healthy eating behavior) or just opposite—worse health status may result in increasing of NRK and NRAs to improve the health status. The present study seems to corroborate the first direction, suggesting that better NRK (and a tendency for more positive NRAs) results in better self-reported health status. It should be noted that our population was generally in quite good health condition, due to, among others, inclusion/exclusion criteria used in the NU-AGE project.

Surprisingly, in this study NRK did not differ between genders. The majority of research in this field indicated that women have higher dietary knowledge than men, regardless the age (Parmenter et al., 2000; Hendrie et al., 2008; Lin et al., 2011; Grunert et al., 2012; Shatenstein et al., 2013). On the contrary, in Taiwanese elderly, men had higher NRK than women, and according to authors it probably resulted from the fact that in Taiwan, elderly women have a lower education level than men (Lin and Lee, 2005). Similarly to our outcome, no differences between women and men in knowledge about fat or fiber were detected in American and Genevan populations (Girois et al., 2001). The differences between women and men in NRK may diminish in some societies because the traditional division of duties in families is reducing (men are also responsible for food shopping and cooking) (Parmenter et al., 2000). Furthermore, the number of people (including men) living alone is increasing forcing them to become more conscious about food and nutrition. Broadly discussed issues of healthy eating in the public space, including media cannot be neglected. Such questions started to appear in magazines for men, not only for women. Taking into account above mentioned considerations more research should be conducted on NRK in women and men, among varied age groups, including older adults as the life expectance constantly increases.

Consequently, more nutrition programs and campaigns dedicated to older adults should be implemented in order to improve their NRK (and NRAs), what in turn may improve their eating habits. Associations between NRK and diet quality were reported in some surveys, like among elderly Canadians (Shatenstein et al., 2013), English adults (Wardle et al., 2000), Belgian adult women (De Vriendt et al., 2009) or Indo-Mauritian adult women (Dunneram and Jeewon, 2013). For example, better NRK was connected with higher consumption of fruit and vegetables and lower intake of fat among English adults (Wardle et al., 2000) and higher intake of fruit and vegetables in Belgian women (De Vriendt et al., 2009). According to data from systematic review, significant positive associations were found between higher nutrition knowledge and a greater intake of cereals or fish, a lower intake of sweetened drinks, a higher intake of fibre or calcium (Spronk et al., 2014). In Indo-Mauritian aged 18–55 years, nutritional knowledge had the highest significant independent effect on diet quality (Dunneram and Jeewon, 2013). It can be speculated that people with higher nutrition knowledge may be more aware of nutritional quality of foods and also be more willing to choose high-quality food products, independently from other socioeconomic factors (Bonaccio et al., 2013). However, in elderly people, some problems with transferring the knowledge into practice may persist (Thomas et al., 2010).

### Nutrition-related attitudes

The results of this study indicate that more positive attitude toward healthy eating was associated with lower BMI and very good or good self-evaluated appetite, as well as higher self-evaluated knowledge about healthy eating. NU-AGE participants belonging to the intervention group showed significantly more positive NRAs than the control group. Among the countries, Italian NU-AGE volunteers had the best NRAs.

Our findings for BMI are in line with a research by Wang et al. (2008) who found out that positive health attitudes were associated with lower BMI in 56–70 years old Australians, even though they calculated BMI on self-reported data of weight and height.

Interestingly, better self-reported appetite was related to more positive NRAs. Shatenstein et al. (2013) found out that elderly Canadian women who reported greater sensations of hunger had better diet quality. Although it is not a direct comparison, it can give some ideas. As authors concluded, feeling hungry (or having a very good appetite) motivates food consumption, what may contribute to better and more conscious food choices. Nevertheless, further research is required to understand and explain the link between appetite and NRAs, and finally with diet quality in older adults.

Similarly to NRK, also NRA score was significantly influenced by the group. The more positive attitudes toward healthy eating patterns detected in NU-AGE intervention group at the follow-up seem logic and may provide some evidence that the NU-AGE project brought positive effects on NRAs (and probably knowledge). Participants during 1 year of NU-AGE dietary intervention received individual dietary advice and selected foods aiming to meet the NU-AGE requirements and had regular (approximately once a month) contacts with dietitians/research nutritionists face-to-face or by telephone, and supported by mail or e-mail (Santoro et al., 2014). Participants that were randomized to control group only received a leaflet with general national dietary guidelines available in each country. As participants were volunteers they all could have quite positive NRAs at baseline, higher than general populations.

Although it was detected only in univariate analysis, significantly higher proportion of women had positive NRAs respect to men. Similarly to NRK, it is commonly assumed that women tend to be more interested in and concerned with healthy eating. Such relationship was found in Taiwanese adults (Lin et al., 2011) but not in Taiwanese elderly people (Lin and Lee, 2005), where men expressed more positive attitudes about general nutrition than women. As discussed above for NRK, this general “truth” for NRAs may also change in the future.

Our study revealed, but only in univariate analysis, that higher proportion of participants who perceived their financial situation as very good had significantly more positive NRAs when compared to subgroups with worse self-evaluated financial situation. In general public it is assumed that healthy eating is also expensive thus people with lower financial resources may think that they are not able to follow dietary recommendations (one out of five statements among NRAs was “I always follow a healthy and balanced diet”). According to some research, high-income or high socio-economic groups found healthy aspects of meal choices or dietary quality more relevant than low-income or low socioeconomic groups (Beydoun and Wang, 2008; Kamphuis et al., 2015).

NRAs are found to be positively correlated with nutrition behavior, more than NRK (Lin et al., 2011). It is in accordance with Knowledge-Attitudes-Behavior Model, which assumes that when health knowledge accumulates, it initiates the changes in attitudes, and when the changes in attitudes accumulate, they result in behavior change (Baranowski et al., 2003).

### Sources of information

For older adults who participated in the present study the main sources of nutrition information were food labels, books and magazines on health, dietitians, doctor's practice, and Internet. These findings are in line with other researches highlighting the importance of health professionals for the dissemination of correct information about nutritional issues. For example, older Americans aged 60–80 years favored health professionals (doctors, dietitians) as nutrition information sources (Schultz et al., 2012). In a population consisting of different age groups, family doctor was the first source of nutrition information, particularly in the elderly (van Dillen et al., 2003). Although dietitian was on the fifth position in the order of most popular sources among the whole population, especially the elderly considered dietitians as a nutrition information source (van Dillen et al., 2003). Interestingly, in the present study, dietitian was significantly more often indicated by older adults in the intervention than in the control group, what may be an effect of regular consultations with dietitian/research nutritionist during the NU-AGE project.

The highest position of food labels among older adults under the present study is a bit surprising, but does not surprise that it was a more popular source among the elderly in intervention than in control group. This can also be a positive effect of the NU-AGE project as participants randomized to intervention group were also advised how to use the food labels for healthy food choices. Food labels were the most common sources of information about functional foods among Canadian older population (Vella et al., 2014). According to van Dillen et al. (2003) food labels were on 8th position, after television, and before other media. Although some discrepancies exist in perceiving food labels as the source of nutrition information, it seems that their role would increase in the future.

For our population, books and magazines on health were a very important source of nutrition information, and this finding was also reported by others (van Dillen et al., 2003; Vella et al., 2014).

The Internet was also quite often indicated in this study. On the contrary, among elderly Americans only 27% reported that they would like to get more nutrition information through the Internet (Schultz et al., 2012). Higher importance of the Internet among older adults in our study may be due to the time difference between both surveys, and thus higher access to and higher popularity of this media (both populations are comparable in demographic characteristics). The Internet was most popular among the youngsters (van Dillen et al., 2003).

Family and friends as a source of nutrition information was not as popular as in other studies, where it was on the first or second place in order (van Dillen et al., 2003; Lin and Lee, 2005).

In turn, three most trusted sources of information (dietitian, books and magazines on health and doctor's practice) show that professionals and professional materials are particularly valuable for surveyed elderly population.

## Limitations of the study

Despite many strengths, this study has some limitations. Firstly, its cross-sectional design may be regarded as a limitation. Some bias may also come from the fact that participants were volunteers in the NU-AGE dietary intervention study and thus could be more interested in nutrition and health than the general population. They could also be of better health condition due to sharp exclusion criteria of the project. Besides, NRK and NRAs were measured after 1 year of follow-up which may also shade the picture and interfere with the final results.

## Conclusions

High NRK and NRAs toward healthy dietary patterns were associated with low BMI and high physical activity level. Therefore, having a good nutrition-related knowledge and attitudes might strongly and positively impact on the health status and quality of life of elderly population. This will offer a great opportunity for policy makers to implement educational programs to counteract the epidemic of obesity. Such programs should be particularly dedicated to elderly people with lower NRK and less positive NRAs, as well as those with a lower socioeconomic status.

## Ethics statement

NU-AGE was approved by the Ethics Committee of the coordinator center: the Independent Ethics Committee of the S. Orsola-Malpighi Hospital Bologna (Italy), and by all the Ethics Committees of all the other four study centers: the South-East 6 Person Protection Committee (France), the Wageningen University Medical Ethics Committee (Netherlands), the National Research Ethics Committee–East of England (UK), and the Bioethics Committee of the Polish National Food and Nutrition Institute (Poland). Written informed consents were collected from all participants prior to their inclusion in the study. The NU-AGE study is registered with clinicialtrials.gov since December 21st 2012 (NCT01754012).

## Author contributions

MJ-B, AK-D, and BP contributed to the conception and design of the current work, data analyses, data interpretation, and drafted the manuscript. CF conceived, designed, initiated and directed NU-AGE. AS coordinated NU-AGE data collection across centers. SH designed the questionnaire for the NRK interviews. AB and LdG designed the dietary intervention study. AK-D, BP, MJ-B, RO, AJ, NM, EC, and RG substantially contributed to the data collection by acquiring or processing data. All authors contributed to interpretation of data, critically revised and approved the final version of this manuscript.

### Conflict of interest statement

The authors declare that the research was conducted in the absence of any commercial or financial relationships that could be construed as a potential conflict of interest.
